# Insulin-like growth factor binding protein-3 has dual effects on gastrointestinal stromal tumor cell viability and sensitivity to the anti-tumor effects of imatinib mesylate *in vitro*

**DOI:** 10.1186/1476-4598-8-99

**Published:** 2009-11-10

**Authors:** Jheri J Dupart, Jonathan C Trent, Ho-Young Lee, Kenneth R Hess, Andrew K Godwin, Takahiro Taguchi, Wei Zhang

**Affiliations:** 1 Department of Pathology, the University of Texas M.D. Anderson Cancer Center, Houston, Texas, USA; 2 Department of Sarcoma Medical Oncology, the University of Texas M.D. Anderson Cancer Center, Houston, Texas, USA; 3 Department of Head and Neck Thoracic Oncology, the University of Texas M.D. Anderson Cancer Center, Houston, Texas, USA; 4 Department of Biostatistics, the University of Texas M.D. Anderson Cancer Center, Houston, Texas, USA; 5 Graduate School of Biomedical Sciences, the University of Texas Health Science Center at Houston, Houston, Texas, USA; 6 Department of Medical Oncology, Fox Chase Cancer Center, Philadelphia, Pennsylvania, USA; 7 Department of Human Health and Medical Science, Kochi University, Nankoku, Kochi, Japan

## Abstract

**Background:**

Imatinib mesylate has significantly improved survival and quality of life of patients with gastrointestinal stromal tumors (GISTs). However, the molecular mechanism through which imatinib exerts its anti-tumor effects is not clear. Previously, we found up-regulation of insulin-like growth factor binding protein-3 (IGFBP3) expression in imatinib-responsive GIST cells and tumor samples. Because IGFBP3 regulates cell proliferation and survival and mediates the anti-tumor effects of a number of anti-cancer agents through both IGF-dependent and IGF-independent mechanisms, we hypothesized that IGFBP3 mediates GIST cell response to imatinib. To test this hypothesis, we manipulated IGFBP3 levels in two imatinib-responsive GIST cell lines and observed cell viability after drug treatment.

**Results:**

In the GIST882 cell line, imatinib treatment induced endogenous IGFBP3 expression, and IGFBP3 down-modulation by neutralization or RNA interference resulted in partial resistance to imatinib. In contrast, IGFBP3 overexpression in GIST-T1, which had no detectable endogenous IGFBP3 expression after imatinib, had no effect on imatinib-induced loss of viability. Furthermore, both the loss of IGFBP3 in GIST882 cells and the overexpression of IGFBP3 in GIST-T1 cells was cytotoxic, demonstrating that IGFBP3 has opposing effects on GIST cell viability.

**Conclusion:**

This data demonstrates that IGFBP3 has dual, opposing roles in modulating GIST cell viability and response to imatinib *in vitro*. These preliminary findings suggest that there may be some clinical benefits to IGFBP3 therapy in GIST patients, but further studies are needed to better characterize the functions of IGFBP3 in GIST.

## Introduction

Gastrointestinal stromal tumors (GISTs) are the most common mesenchymal tumors of the digestive tract. GIST pathogenesis is most frequently attributed to gain-of-function mutations in the receptor tyrosine kinase *KIT*; however, activating mutations in *platelet derived growth factor receptor-α *(*PDGFRA*) have been observed in GISTs with wild-type *KIT *[1]. This trend of oncogenic KIT or PDGFRA expression is observed in approximately 85% of tumors [2,3]. Traditionally, surgery was the only successful therapeutic strategy; however, patients with unresectable or metastatic disease survived only a median of 18-24 months after diagnosis [4,5]. Those patients with widespread metastatic disease have an estimated 9 month overall survival [6]. The development of the selective kinase inhibitor imatinib mesylate (also known as Gleevec) has dramatically altered the treatment strategies for GIST and other cancers.

An ATP mimetic, imatinib competitively occupies the ATP binding pocket of target kinases, thereby preventing their activation [7]. Although designed to specifically target PDGFR, imatinib also effectively inhibits KIT and Abl kinases, which have structurally similar ATP binding pockets [8]. Thus, imatinib is successful as a targeted therapy in GIST through inhibition of KIT or PDGFRA, and in other cancers, including Philadelphia chromosome-positive chronic myelogenous leukemias through inhibition of Bcr-Abl [9]. Clinical studies with imatinib have reported objective response rates of 50-70% and an estimated median survival of 57 months in patients with advanced GIST [10]. However, some GIST patients fail to respond or become resistant to imatinib therapy [9,11]. Therefore, to further improve GIST patient survival, it is imperative to gain a better understanding of the underlying molecular mechanisms of imatinib-induced GIST cell cytotoxicity.

In a previous study to determine how imatinib exerts its anti-tumor effects, we demonstrated that insulin-like growth factor binding protein-3 (IGFBP3) expression is up-regulated after imatinib treatment in the imatinib-responsive GIST cell line GIST882 as well as KIT-expressing tumor samples [12]. IGFBP3, a member of the insulin-like growth factor binding protein family, is a multifunctional protein that directly binds and regulates the mitogenic and anti-apoptotic actions of the insulin-like growth factors (IGFs) [13]. IGFBP3 also has IGF-independent growth inhibitory and pro-apoptotic effects, which may be mediated through cell surface [14] or nuclear receptors [15-17]. Furthermore, expression of IGFBP3 is induced by a number of growth inhibitory and pro-apoptotic agents, including p53 [18,19], TGF-β [20,21], retinoids [20], TNF-α [22], vitamin D [23], and celecoxib [24], suggesting that IGFBP3 may, in part, mediate their anti-tumor effects.

Having identified IGFBP3 as a candidate imatinib-targeted gene, we sought to determine whether IGFBP3 directly mediates the cytotoxicity of imatinib in GIST cells. In this study, we manipulated IGFBP3 levels in two imatinib-responsive GIST cell lines and observed cell viability after drug treatment. We found that IGFBP3 down-regulation in GIST882 cells resulted in a loss of cell viability and partial resistance to imatinib. In contrast, IGFBP3 overexpression was cytotoxic but did not enhance or abrogate the cytotoxic effects of imatinib in GIST-T1 cells. Thus, IGFBP3 has cell-dependent effects on GIST cell viability and in mediating imatinib response.

## Results

### Heterogeneous induction of IGFBP3 after imatinib in GIST cell lines

**Figure 1:**
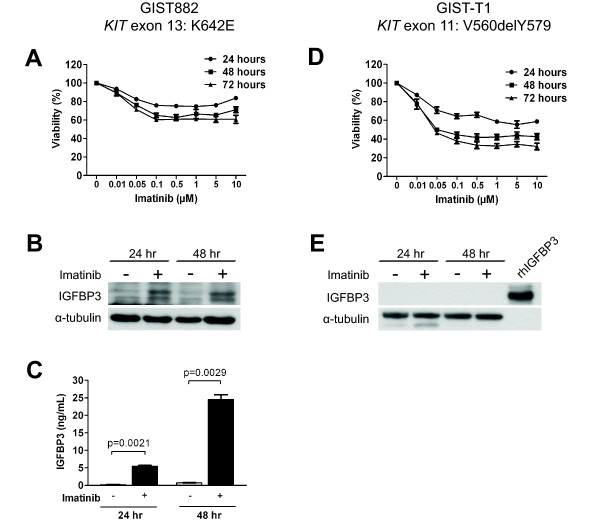
**Imatinib-induced IGFBP3 expression is heterogenous in GIST cells**. GIST882 (A) or GIST-T1 (D) cells were treated with different doses of imatinib for 24, 48, or 72 hours and then analyzed with the MTS assay to determine cell viability. After treatment with 1 μM imatinib for 24 or 48 hours, whole cell lysates isolated from GIST882 (B) or GIST-T1 (E) were analyzed for IGFBP3 expression by immunoblotting, or (C) conditioned medium from GIST882 cells analyzed for IGFBP3 levels by ELISA. *rhIGFBP3*, recombinant human IGFBP3.

To study the role of IGFBP3 in GIST, we used two available GIST cell lines: GIST882 and GIST-T1. The GIST882 cell line harbors a missense mutation in *KIT *exon 13 (K642E) affecting the kinase domain. Imatinib treatment of GIST882 cells results in a loss of viability of up to 40% with doses as low as 0.1 μM, but the response appeared to reach a plateau, as higher doses of imatinib had no additional effect on cell viability (Figure 1A). Imatinib treatment induced endogenous IGFBP3 expression by 24 hours after treatment, and the induction was maintained at 48 hours post-treatment (Figure 1B). Because IGFBP3 is a secreted protein that can be re-internalized into the cell [25], we used an ELISA to determine if elevated levels of IGFBP3 were also present in the cell culture medium after imatinib exposure. As shown in Figure 1C, imatinib treatment significantly increased the concentration of IGFBP3 in the conditioned medium, consistent with imatinib-induced IGFBP3 up-regulation and secretion by GIST882 cells. The GIST-T1 cell line has an in-frame deletion of 57 nucleotides in *KIT *exon 11 (V560delY579) affecting the juxtamembrane regulatory domain. Imatinib treatment resulted in a loss of viability of more than 60% at a concentration of 0.5 μM, and the observed IC_50 _was 0.05 μM at 48 and 72 hours (Figure 1D). In contrast to GIST882 cells, GIST-T1 cells did not have detectable basal levels of IGFBP3, nor did imatinib treatment induce IGFBP3 expression (Figure 1E). This data was a preliminary indicator that IGFBP3 induction is not required for GIST-T1 cell response to imatinib. However, imatinib-induced IGFBP3 expression in the relatively more resistant GIST882 cells suggested that IGFBP3 might contribute to a resistance phenotype.

### IGFBP3 has cell-dependent effects on viability in GIST cells

**Figure 2:**
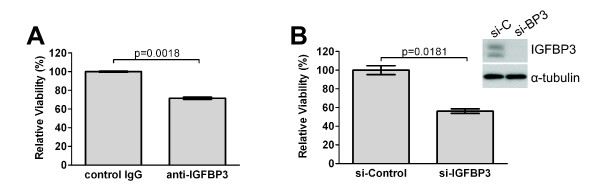
**Effect of IGFBP3 knockdown or neutralization on GIST882 cell viability**. (A) GIST882 cells were treated with IGFBP3 blocking antibody (anti-IGFBP3) or control IgG (4 ug/mL) for 48 hours before cell viability was measured with the MTS assay. (B) GIST882 cells were transfected with siRNA duplexes specific to IGFBP3 (si-IGFBP3) or mismatch sequence (si-Control) and subsequently treated with imatinib (1 μM) for 48 hours. Cell viability was assessed with the MTS assay. Immunoblotting shows IGFBP3 expression after si-Control (si-C) or si-IGFBP3 (si-BP3) transfection.

**Figure 3:**
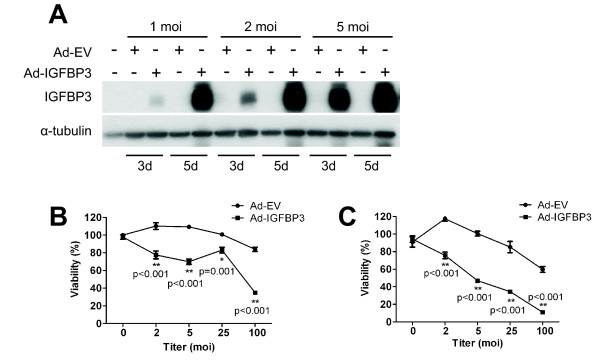
**Effect of IGFBP3 overexpression on GIST-T1 cell viability**. (A) Whole cell lysates isolated from GIST-T1 cells three or five days after mock infection or infection with indicated titers (moi) of adenovirus expressing IGFBP3 (Ad-IGFBP3) or empty vector (Ad-EV) were analyzed by immunoblotting for IGFBP3 expression. Viability of GIST-T1 cells mock infected or infected with indicated titers of Ad-EV or Ad-IGFBP3 was analyzed 3 days (B) or 5 days (C) post-infection with the MTS assay. Asterisks (*) denote significant differences in cell viability at the indicated doses of Ad-EV vs. Ad-IGFBP3.

Before we evaluated whether IGFBP3 modulates GIST cell response to imatinib, we first sought to determine what effects IGFBP3 itself has in the two GIST cell lines. Because GIST882 cells have detectable levels of endogenous cellular and secreted IGFBP3, we down-modulated IGFBP3 using either a blocking antibody to sequester secreted IGFBP3 or an siRNA knockdown approach. Both IGFBP3 neutralization using a blocking antibody (Figure 2A) and knockdown using RNA interference (Figure 2B) resulted in a significant loss of cell viability, suggesting that IGFBP3 is required for GIST882 cell survival. In contrast, as GIST-T1 cells have no detectable endogenous IGFBP3, we overexpressed IGFBP3 using an adenoviral gene expression system. Infection with an adenoviral vector expressing IGFBP3 (Ad-IGFBP3) but not empty vector (Ad-EV) resulted in high, sustainable levels of IGFBP3 (Figure 3A). IGFBP3 overexpression in GIST-T1 cells resulted in a dose-dependent loss of cell viability as observed 3 (Figure 3B) and 5 days (Figure 3C) post adenoviral infection. These results show that IGFBP3 overexpression reduces cell viability in GIST-T1 cells. Collectively, our data demonstrate that IGFBP3 has dual, opposing cell line-dependent effects on GIST cell survival.

### IGFBP3 modulation alters GIST cell sensitivity to imatinib in a cell-dependent manner

**Figure 4:**
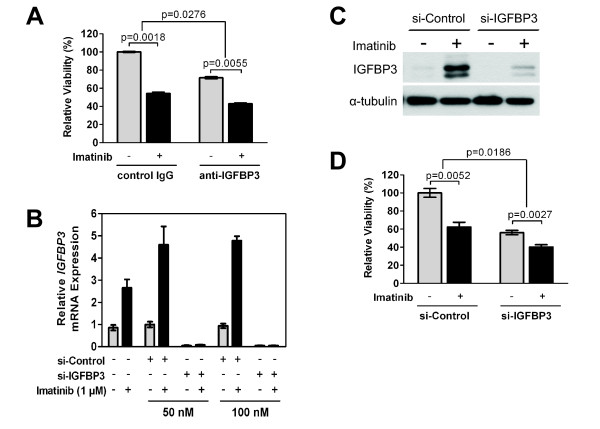
**Effect of IGFBP3 neutralization or knockdown on GIST882 response to imatinib**. (A) Cells were treated simultaneously with imatinib (1 μM) and anti-IGFBP3 or control IgG (4 μg/mL) for 48 hours, and viability was assessed with the MTS assay. (B) RNA was extracted from ST882 cells after transfection with of siRNA (50 or 100 nM) and imatinib (1 μM) for 48 hours. After the reverse transcriptase reaction, *IGFBP3 *mRNA levels were quantified by real-time PCR. (C)Whole cell lysate isolated after siRNA (50 nM) transfection and imatinib (1 μM) treatment were analyzed for IGFBP3 expression by immunoblotting. (D) Viability after siRNA (50 nM) transfection and imatinib treatment (1 μM) was assessed by MTS.

The above studies show a complex pattern of IGFBP3 function in regulating GIST cell survival. To determine whether IGFBP3 contributes to GIST cell response or resistance to imatinib, we modulated IGFBP3 protein levels in GIST882 and GIST-T1 cells and assayed for potential changes in imatinib sensitivity. Because IGFBP3 is a secreted protein that can be re-internalized into the cell [25], we first used an IGFBP3 blocking antibody to sequester secreted IGFBP3 in the culture medium. GIST882 cells were simultaneously treated with imatinib and different concentrations of IGFBP3 blocking antibody (anti-IGFBP3) or control IgG and viability assessed with the MTS assay. Treatment with anti-IGFBP3 alone significantly reduced cell viability (p = 0.0018) relative to control IgG (Figure 4A). To determine the effects IGFBP3 neutralization on imatinib sensitivity, MTS data were analyzed with a two-way ANOVA (interaction: p < 0.0001). As shown in Figure 4A, imatinib significantly reduced viability in both cells treated with anti-IGFBP3 or control IgG; however imatinib was significantly less cytotoxic in the presence of anti-IGFBP3. Because IGFBP3 is also localized intracellularly, we used RNA interference to suppress drug-induced IGFBP3 expression. As shown in Figure 4B, si-IGFBP3 effectively reduced *IGFBP3 *mRNA levels in untreated cells and also prevented *IGFBP3 *induction after imatinib exposure. Loss of IGFBP3 protein expression after imatinib was also observed (Figure 4C). Using this siRNA, we investigated whether inhibiting IGFBP3 expression altered GIST882 cell sensitivity to imatinib. IGFBP3 knockdown itself was cytotoxic to GIST882 cells (p = 0.018) (Figure 4D). We further analyzed the effects of IGFBP3 expression on GIST882 cell sensitivity to imatinib with a two-way ANOVA (interaction: p = 0.0243). As demonstrated in Figure 4D, imatinib treatment significantly reduced viability in si-IGFBP3 or si-Control transfected cells; however, cells with reduced IGFBP3 expression were partially resistant to the cytotoxic effects of imatinib. Taken together, this data suggests that IGFBP3 sensitizes GIST882 cells to the anti-tumor effects of imatinib.

**Figure 5:**
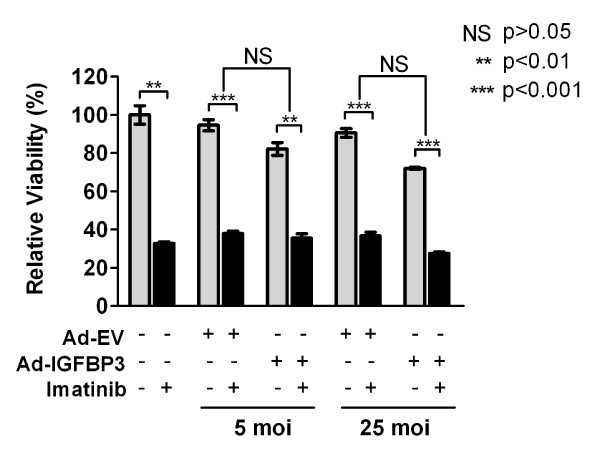
**Effect of IGFBP3 overexpression on GIST-T1 cell sensitivity to imatinib**. GIST-T1 cells were mock infected or infected with indicated titers (moi) of Ad-IGFBP3 or Ad-EV. The following day, infected cells were exposed to imatinib (0.075 μM) for 48 hours. Viability was assessed with the MTS assay. *NS*, not significant.

In contrast to GIST882 cells, GIST-T1 cells have no endogenous IGFBP3 expression and there was no induction of IGFBP3 after imatinib treatment. Therefore, this IGFBP3-negative cell line provided us a system to examine the effects of IGFBP3 on imatinib response using a gain-of-function approach. To test whether IGFBP3 expression altered GIST-T1 sensitivity to imatinib, cells infected with Ad-IGFBP3 or Ad-EV or mock infected were subsequently analyzed for changes in cell viability after imatinib. Ad-IGFBP3 infection alone was cytotoxic relative to Ad-EV at 25 moi (p = 0.0024) (Figure 5). Analyzing imatinib sensitivity after IGFBP3 overexpression with a two-way ANOVA (interaction: p = 0.0009), we observed that imatinib significantly reduced viability in cells infected with Ad-IGFBP3 or Ad-EV. Furthermore, IGFBP3 overexpression did not significantly alter imatinib sensitivity in GIST-T1 cells (Figure 5). Although IGFBP-3 is cytotoxic to GIST-T1 cells, our data suggests that IGFBP3 does not mediate GIST-T1 response to imatinib.

## Discussion

In this study, we examined the potential role of IGFBP3 as a mediator of the therapeutic effects of imatinib mesylate in GISTs. Our previous studies showed that IGFBP3 is up-regulated after imatinib treatment in a responsive GIST cell line (GIST882), and we provide evidence that IGFBP3 does indeed partially mediate GIST882 cell response to imatinib *in vitro*. In contrast, IGFBP3 has no effect on imatinib sensitivity in the responsive GIST-T1 cell line, which has no detectable endogenous IGFBP3 levels before or after imatinib exposure. Further, our studies, using both gain-of-function and loss-of-function approaches, reveal that IGFBP3 is an important modulator of cell viability in GISTs, but the effect is cell-dependent. Similar to what has been reported for epithelial cancers [23,26-34], IGFBP3 also manifests dual functions on cell survival in GIST, a mesenchymal cancer.

Up-regulation of IGFBP3 has been observed in response to a variety of anti-cancer agents [18-23], including celecoxib [24]. In addition, IGFBP3 potentiates the action of paclitaxel [35] and sensitizes cancer cells to the cytotoxic effects of gefitinib [36] and other chemotherapeutic agents [37]. Because we observed IGFBP3 expression in GIST in response to imatinib [12], we hypothesized that IGFBP3 would mediate its anti-tumor effects. After manipulating IGFBP3 levels in two GIST cell lines, we observed a modulating effect on response in GIST882, suggesting that the induction of IGFBP3 is a significant, specific response to imatinib-induced stress. Failure to observe a similar response in GIST-T1 suggested that GIST-T1 cells are insensitive to IGFBP3. However, additional studies showed that IGFBP3 regulates GIST cell viability with opposing effects. Overexpression of IGFBP3 in GIST-T1 cells, which have no detectable endogenous IGFBP3 expression before or after imatinib, results in a loss of cell viability, demonstrating that IGFBP3 has growth inhibitory effects in this cell line. In contrast, we expected that the loss of IGFBP3 by neutralization or knockdown in GIST882 cells, which have increased IGFBP3 expression after imatinib, would have a protective effect on cell viability. However, our data shows that IGFBP3 down-modulation is cytotoxic, demonstrating that IGFBP3 is necessary for cell viability. Thus, in GIST882, IGFBP3 has two distinct roles, which may be attributed to a dose-dependent mechanism. Dual functions of IGFBP3 have been reported previously in cancers of the renal cells [26,27], esophagus [28,29], breast [30,31], colon [32,33], and prostate [23,34], as well as in endothelial cells [38]. The mechanism that determines the final outcome of IGFBP3 action is not well understood, though some studies suggest a role for post-translational modification [39], localization within specific cellular compartments [25,40], extracellular matrix composition [41], or binding partner interaction [15,42,43]. Despite its dual effects on GIST cell viability, IGFBP3 appears to exert its effects through a KIT-independent mechanism, as imatinib-induced KIT inactivation has no effect on IGFBP3-mediated loss of cell viability in either GIST882 or GIST-T1 cells.

IGFBP3 expression is lost in many cancer cells [44-46], and reintroduction of the protein often results in cell death [21,46,47]. Similarly, our results show that IGFBP3 expression is not detectable in GIST-T1 cells but overexpression leads to loss of cell viability. Indeed, the growth inhibitory and pro-apoptotic effects of IGFBP3 are well established in a variety of *in vitro *and *in vivo *cancer models. On the other hand, IGFBP3 also has growth stimulatory effects [29,33,34,38,41,48], depending on the cell type and context. Further, increased IGFBP3 expression has also been linked to renal cell carcinoma [27], breast cancer [31,49], and metastatic melanoma [50], suggesting that IGFBP3 may contribute to tumorigenesis or disease progression. Here, we report that GIST882 cells, which have detectable IGFBP3 protein expression, require IGFBP3 for cell viability, confirming the notion that IGFBP3 may facilitate cancer cell proliferation and survival. Complete understanding of IGFBP3 requires investigations of its binding partners, post-translational modifications, and signal transduction pathways *in vitro *and *in vivo*.

One possible pathway through which IGFBP3 may exert its effects in GISTs is the IGF pathway. A number of recent studies have explored the IGF axis for prognostic and therapeutic value in GISTs. Braconi and colleagues reported that expression of IGF-1 and IGF-2 is correlated with poor prognosis and relapse, and that IGF-1R expression was strong in all cases [51]. Furthermore, Tarn and colleagues reported that knockdown of IGF-1R was cytotoxic in GIST-T1 cells [52]. IGFBP3 is the most abundant IGF binding protein in the circulation and is responsible for a majority of IGF transport [53]. Because IGFBP3 has intrinsic IGF-binding activity that can act to sequester IGF from its cognate receptor [13], it is possible that using IGFBP3 as a therapeutic agent would be useful to GIST patients with abnormal IGF expression or IGF-dependent IGF-1R activation. Furthermore, if IGFBP3 is indeed acting through an IGF-dependent mechanism, a difference in the expression levels of IGF or IGF-1R or increased sensitivity to IGF might contribute to the differential IGFBP3-induced effects on cell viability and imatinib response in GIST882 or GIST-T1. Additional studies are needed to determine IGF and IGF-1R expression levels and IGF sensitivity in GIST cell lines and to further examine whether IGFBP3 functions through an IGF-dependent or IGF-independent mechanism in GIST.

In addition to its direct effects on cancer cells, IGFBP3, as a secreted protein, may also have paracrine effects on the tumor environment. Recent studies report that IGFBP3 regulates endothelial cell survival [54] and suppresses angiogenesis [55,56]. Thus, it is possible that IGFBP3 further modulates the viability of GIST cells or alters their response to imatinib by targeting endothelial cells or other important cell types, such as macrophages, in the tumor microenvironment. However, the present study is limited to an *in vitro *cell culture system. Mouse model studies are needed to further investigate whether the effects of IGFBP3 extend to the GIST microenvironment.

## Conclusion

Here, we present evidence that IGFBP3 has dual, opposing effects on GIST cell viability and that IGFBP3 partially mediates the anti-tumor effects of imatinib mesylate in some GISTs *in vitro*. Further studies are needed to elucidate the mechanisms of IGFBP3 action and to evaluate IGFBP3 as a potential therapeutic agent or target in GISTs.

## Methods

### Reagents

Imatinib mesylate (Gleevec™, Glivec^®^, CGP57148, formerly STI-571) was obtained from Novartis Oncology (East Hanover, NJ). For drug treatment, imatinib was prepared as a 10 mM solution in sterile water and subsequently filter-sterilized using 0.45 μm filters (Millipore).

### Cell Culture

The GIST882 cell line was kindly provided by Dr. Jonathan Fletcher (Dana-Farber Cancer Institute, Boston, MA) and was described previously [57]. The GIST-T1 cell line was described previously [58]. Cells were cultured in Dulbecco's minimal essential medium high glucose supplemented with 10% fetal bovine serum and maintained at 37°C in a humidified incubator with 5% CO_2_.

### IGFBP3 neutralization

The non-internalizable IGFBP3 blocking antibody, goat polyclonal anti-IGFBP3, was acquired from Diagnostic Systems Laboratories (DSL-R00536, Webster, TX). The corresponding anti-goat IgG (Vector Laboratories, Inc., Burlingame, CA) was used as a control. GIST882 cells were seeded at 4 × 10^5 ^cells/well in 6-well plates and subsequently treated with antibody or IgG alone or in the presence of imatinib for 48 hours before being assayed for changes in cell viability.

### RNA interference

Knockdown experiments were performed using Ambion *Silencer *pre-designed siRNA (Ambion, Austin, TX). For *IGFBP3 *silencing, the selected siRNA (ID #144575) targets exon 5 and its sequence is given below.

Sense - 5' CGAAGCUUAUUUCUGAGGAtt 3'

Antisense - 5' UCCUCAGAAAUAAGCUUCGtc 3'

A non-silencing mismatch siRNA, *Silencer *Negative Control #1 (AM4635), was used as a negative control. Transfection of siRNA duplexes was performed with Ambion *Silencer *siPORT NeoFX reagent (AM4510) according to the manufacturer's instructions. Briefly, siRNA was diluted in serum-free minimum essential medium supplemented with non-essential amino acids and NeoFX reagent before mixing with 8 × 10^3 ^cells/well in 96-well plates or 2 × 10^5 ^cells/well in 6-well plates. The final concentration of siRNA in the solution was 50 nM. After 48 hours, cells were exposed to imatinib for an additional 48 hours before being assayed for changes in cell viability.

### ELISA

GIST882 cells (3 × 10^6^) were treated with imatinib for 24 or 48 hours. After treatment, the conditioned medium was collected and briefly centrifuged to remove the floating cells and cellular debris. Aliquots (50 μL) of the supernatant were analyzed for the presence of IGFBP3 using the Human IGFBP3 Quantikine ELISA kit (#DGB300) from R&D Systems (Minneapolis, MN) according to the manufacturer's instructions.

### Adenovirus-mediated gene transduction

Adenoviral vectors expressing IGFBP3 (Ad-IGFBP3) or empty vector (Ad-EV) were described previously [47]. For infection of GIST-T1, 8 × 10^3 ^cells/well were seeded to 96-well plates or 2 × 10^5 ^cells/well were seeded to 6-well plates and allowed to adhere overnight. The following day, cells were mock-infected or infected with the indicated titers of Ad-IGFBP3 or Ad-EV for 2 hours and then incubated in complete medium. The next day, cells were exposed to imatinib for 48 hours before being assayed for changes in cell viability.

### MTS assay

Cell viability was assessed using the 3-(4,5-dimethylthiazol-2-yl)-5-(3-carboxymethoxyphenyl)-2-(4-sulfophenyl)-2H-tetrazolium bromide (MTS) (Promega Corporation, Madison, WI) assay as described previously [59].

### Real-time PCR

Total RNA was isolated from GIST882 cells after siRNA transfection using the Qiagen RNeasy Mini Kit (Valencia, CA). After reverse transcription, real-time PCR was performed as described previously [12]. Primers for IGFBP3 (assay ID Hs00181211_m1) and the endogenous control cyclophilin A (gene name PPIA, #4326316E), as well as TaqMan Universal PCR Master Mix (#4324018) were obtained from Applied Biosystems (Foster City, CA).

### Antibodies

Primary antibodies used include the following: anti-IGFBP3 (DSL-R00536, 1:3000) from Diagnostic Systems Laboratories (Webster, TX) and anti-α-tubulin (T5168, 1:5000) from Sigma (St. Louis, MO). Secondary antibodies used include anti-goat (sc-2020, 1:1000) from Santa Cruz (Santa Cruz, CA) and anti-mouse (#7076, 1:1000) from Cell Signaling (Danvers, MA).

### Immunoblotting

Cells were washed in cold PBS and then incubated in dispersal buffer (PBS + 1 mM EDTA, pH 8) to begin dissociation. Cells were scraped gently, collected, and centrifuged at 2000 rpm for 10 min before resuspension in cold lysis buffer containing 25 mM HEPES, pH 7.5, 150 mM NaCl, 1% NP-40, 10 mM MgCl_2_, 1 mM EDTA, and 10% glycerol (Upstate, Lake Placid, NY) and supplemented with protease inhibitor cocktail and phosphatase inhibitor cocktails 1 and 2 (1:100) (Sigma, St. Louis, MO). After incubation on ice for 30 minutes and subsequent centrifugation at 14,000 rpm at 4°C for 15 minutes, supernatants were collected and protein concentration determined using the Bio-Rad Protein Assay (Bio-Rad, Hercules, CA). Protein (40 μg) was resolved by SDS-polyacrylamide gel (8-12%) electrophoresis, followed by transfer to polyvinylidene fluoride (PVDF) membranes. Membranes were blocked for 1 hour in Tris-buffered saline (TBS) containing 0.05% Tween 20 (TBS-T) and 5% nonfat dry milk and probed overnight with primary antibody at 4°C. After washing several times in TBS-T, membranes were probed with the corresponding horseradish peroxidase (HRP)-conjugated secondary antibody for 1 hour at room temperature. Membranes were washed several times in TBS-T and protein signal detected using ECL (Amersham Biosciences, Piscataway, NJ) or SuperSignal chemiluminescence reagent (Pierce, Rockford, IL).

### Statistics

Values given are mean ± SEM. Data was analyzed with Student's t-test or two-way ANOVA where indicated. P-values less than 0.05 were considered significant.

## Competing interests

The authors declare that they have no competing interests.

## Authors' contributions

All authors participated in the design of the study. JJD performed all experiments and drafted the manuscript. All authors read, revised, and approved the final manuscript.
